# Supplementary material for: Rotation-Direction-Dependent Mechanism of the Inhibitor Protein IF1 for Mitochondrial ATP Synthase from Atomistic Simulations
Source: JACS Au. 2025 May 27;5(6):2654–65. doi: 10.1021/jacsau.5c00261 (PMC12188409; doi:10.1021/jacsau.5c00261)
Supplement: Supplementary file 1 [file au5c00261_si_001.pdf]

Supporting information for

Rotation-Direction-Dependent Mechanism of the Inhibitor Protein IF_1 for Mitochondrial ATP Synthase from Atomistic Simulations

Ryohei Kobayashi[1], Kei-ichi Okazaki[1,2,*]

This PDF file includes:

- Materials and Methods
- Figs. S1 to S9
- Table. S1
- References

[1]*Research Center for Computational Science, Institute for Molecular Science, National Institutes of Natural Sciences, Okazaki, Aichi 444-8585, Japan*
[2] *Graduate Institute for Advanced Studies, SOKENDAI, Okazaki, Aichi 444-8585, Japan*

*Corresponding author: Kei-ichi Okazaki
Email: keokazaki@ims.ac.jp

Materials and methods

Torque-applying simulation

In this study, the rotational angles of the γ-subunit, represented by its Cα atoms, were computed using a displacement vector projected onto a plane perpendicular to the rotation axis[1]. The displacement vector $\vec{s_i}$ for each residue i was defined as

$$\vec{s_i} = \vec{r_i} - \overrightarrow{r_{CM}} - [(\vec{r_i} - \overrightarrow{r_{CM}}) \cdot \vec{v}]\, \vec{v} \quad \text{(Eq.1)}$$

where, $\vec{r_i}$ is the positional vector of residue i, $\overrightarrow{r_{CM}}$ is the center of mass of the Cα atoms, and the $\vec{v}$ represents the rotation axis (the z-axis in the current study). This projection ensures that the displacement vector lies within the plane perpendicular to the rotation axis. The rotational angle φ_i for each residue i was then calculated as the angle between the initial displacement vector $\overrightarrow{s_i^0}$ and the current displacement vector $\vec{s_i}$, using the following expression:

$$\varphi_i = \varphi_i^0 + \cos^{-1}\left(\frac{\overrightarrow{s_i^0} \cdot \vec{s_i}}{\left|\overrightarrow{s_i^0}\right| \left|\vec{s_i}\right|}\right) \quad \text{(Eq.2)}$$

where φ_i^0 represents the initial rotational angle. To eliminate the overall translation of the γ-subunit, the center of mass $\overrightarrow{r_{CM}}$ was subtracted from the positional vectors.

In the simulations, distance-weighted averages of the cosine and sine of the rotational angles were controlled. The weight is the length of the displacement vectors $\vec{s_i}$. The distance-weighted average cosine and sine of the rotational angle were given by:

$$\overline{\cos \Delta\varphi} = \frac{\sum_i |\vec{s_i}| \cos(\varphi_i - \varphi_i^0)}{\sum_i |\vec{s_i}|}, \overline{\sin \Delta\varphi} = \frac{\sum_i |\vec{s_i}| \sin(\varphi_i - \varphi_i^0)}{\sum_i |\vec{s_i}|} \quad \text{(Eq.3)}$$

with,

$$\cos(\varphi_i - \varphi_i^0) = \frac{\overrightarrow{s_i^0} \cdot \vec{s_i}}{\left|\overrightarrow{s_i^0}\right| |\vec{s_i}|}, \sin(\varphi_i - \varphi_i^0) = \frac{\left(\vec{v} \times \overrightarrow{s_i^0}\right) \cdot \vec{s_i}}{\left|\overrightarrow{s_i^0}\right| |\vec{s_i}|} \quad \text{(Eq.4)}$$

The torque potential $V(t)$ was defined as:

$$V(t) = \frac{\kappa}{2}\left\{\left(\overline{\cos \Delta\varphi} - \cos \omega t\right)^2 + \left(\overline{\sin \Delta\varphi} - \sin \omega t\right)^2\right\} \quad \text{(Eq.5)}$$

where, κ is the force constant (10^4, 10^5 and 10^6 kcal・mol^{-1}・rad^{-2}), ω is the angular velocity of the rotation (1 °/ns). For the analysis, the rotation angle of the γ subunit (Fig. 2C, 2D, and Fig. S2) was calculated as:

$$\Delta\varphi = \frac{\sum_i |\vec{s_i}| (\varphi_i - \varphi_i^0)}{\sum_i |\vec{s_i}|} \quad \text{(Eq.6)}$$

Work and torque estimation

The non-equilibrium work at time *t* by the torque potential (Fig. 2E, 2F, and Fig. S3) is defined as the previous paper [2]:

$$W_t = \int_0^t \frac{\partial V(t')}{\partial t'} dt' \tag{Eq.7}$$

We obtain

$$\begin{aligned} \frac{\partial V(t)}{\partial t} &= \kappa\{\omega\left(\overline{\cos\Delta\varphi} - \cos\omega t\right)\sin\omega t \ - \omega(\overline{\sin\Delta\varphi} - \sin\omega t)\cos\omega t\} \\ &= \left[\vec{r}\times\vec{F}\right]_z \cdot \omega \end{aligned} \tag{Eq.8}$$

Here, we define

$$\vec{r} = \begin{pmatrix} \cos\omega t \\ \sin\omega t \\ 0 \end{pmatrix}, \vec{F} = \begin{pmatrix} -\kappa\left(\overline{\cos\Delta\varphi} - \cos\omega t\right) \\ -\kappa(\overline{\sin\Delta\varphi} - \sin\omega t) \\ 0 \end{pmatrix} \tag{Eq.9}$$

Then,

$$\frac{\partial V(t)}{\partial t} = \left[\vec{r}\times\vec{F}\right]_z \cdot \omega \ = \frac{dW}{dt} \tag{Eq.10}$$

where $\left[\vec{r}\times\vec{F}\right]_z$ represents the applied torque in the simulations (Fig. S4).

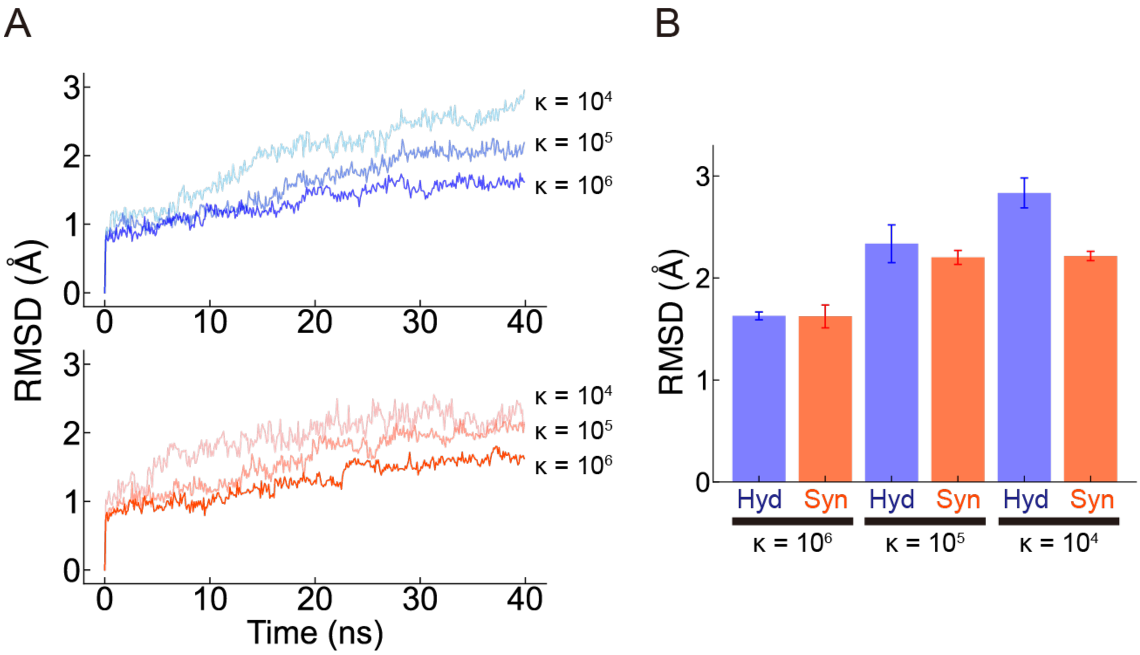

Fig. S1. Twisting of the γ subunit during the torque-applying simulation.
(A) Root-mean-square deviation (RMSD) plot during 40 ns simulation of CCW direction (upper, blue) and CW direction (lower, orange). (B) The average RMSD value at 40 ns. Bars represent SD from three independent simulations.

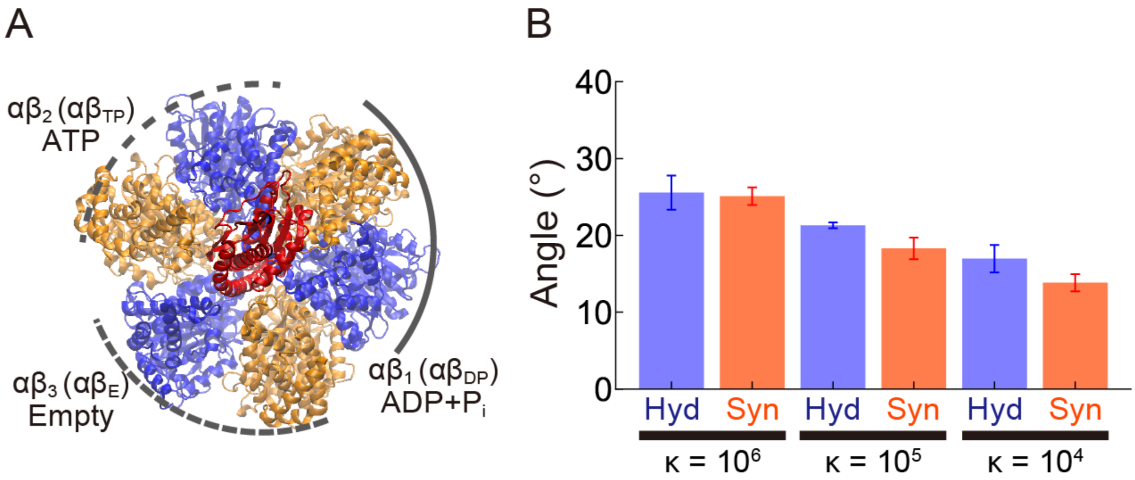

Fig. S2. Simulations of IF_1-free *b*MF_1.
(A) The top view of the F_1 structure with the initial bound nucleotides. The δ and ε subunits are omitted in this figure. (B) The final core rotation from the 40 ns simulation. Values represent the mean, and error bars represent SD, estimated from 3 independent simulations.

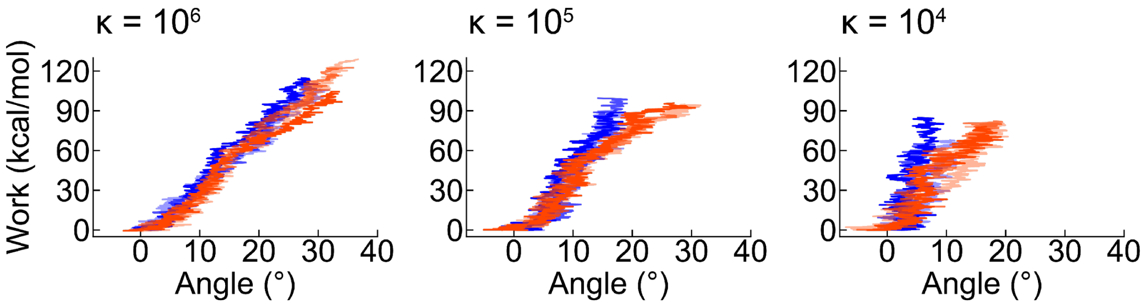

Fig. S3. Non-equilibrium work along the core angle with three different κ values.
Three simulation trajectories are shown in gradation. The plots show that the increase of non-equilibrium work is steeper for CCW rotation (blue; the hydrolysis direction) than CW rotation (orange; the synthesis direction) along the core angle.

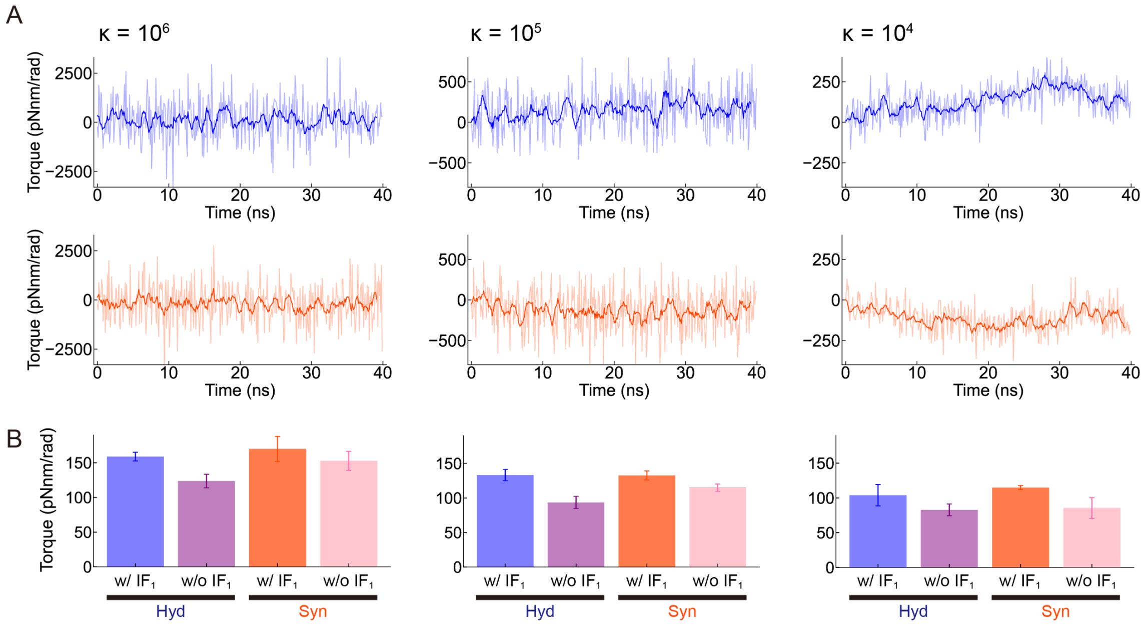

Fig. S4. Torque estimation during simulations.

(A) The torque plots of the CCW (blue; *Upper*) and the CW (orange; *Lower*) simulations of the IF_1-bound F_1. Positive and negative are defined as CCW and CW directions, respectively. The raw data and the running average are represented by light and dark colors, respectively. (B) The average torque values from three independent simulations are plotted. The values in the CW direction were converted to positive ones. Values and error bars represent the mean and the SD, respectively.

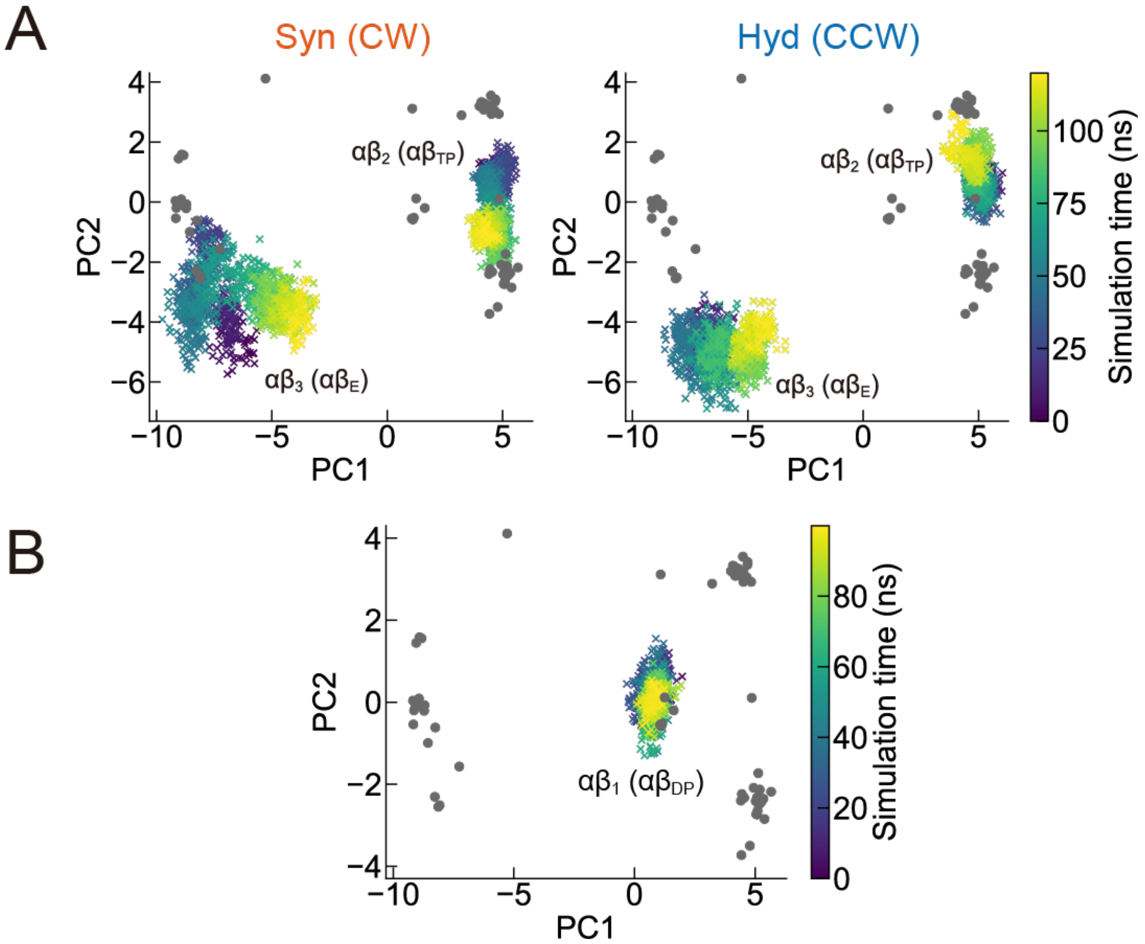

Fig. S5. Conformational changes of each αβ pair projected onto the PC1-PC2 plane, determined by PCA of the X-ray crystal structures shown in gray dots (Fig. 1B).
(A) Conformational changes of the $αβ_2$ ($αβ_{TP}$) and $αβ_3$ ($αβ_E$) pair upon the 120° rotation with the CW (left) and CCW (right), whereas that of the IF_1-bound $αβ_1$($αβ_{DP}$) is presented in Fig. 3A and 3B. (B) Conformational change of the IF_1-bound $αβ_1$ ($αβ_{DP}$) during 100 ns of the equilibrium run before the torque-applying simulation.

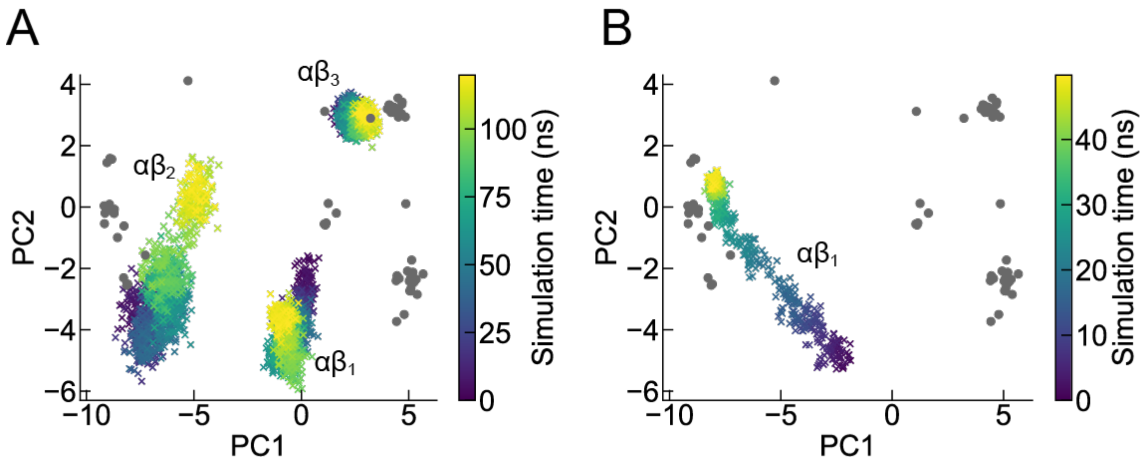

Fig. S6. Conformational change of the αβ pairs after CW 120° rotation.
(A) The CW γ rotation from 120° to 240°. (B) The targeted MD at 240°. The gray dots correspond to the X-ray crystal structures (Fig. 1B).

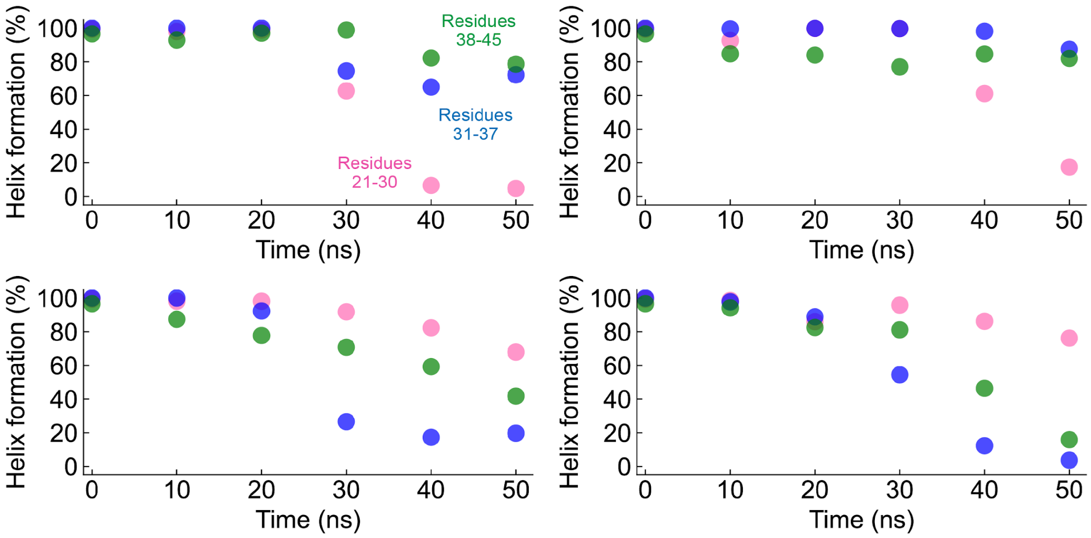

Fig. S7. Time-dependent long helix deformation in four independent runs during the targeted MD at CW 240°.

The long helix was divided into three parts: residues 21-30 (pink), 31-37 (blue), and 38-45 (green). The helix formation at time 0 was estimated from the simulation result before the torque-applying simulation. The left upper one is the same as Fig. 4C. The upper two results show that the deformation of the long helix occurred at the entrance of the long helix (pink), while the lower two results show that the deformation occurred at the second half of the long helix (blue).

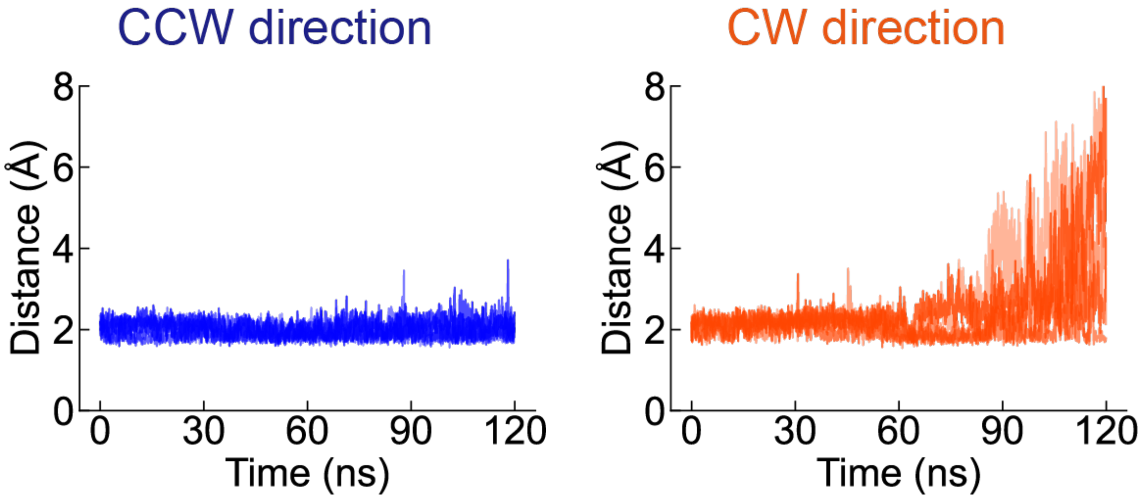

Fig. S8. Minimum distance between the short helix and the γ subunit during 120° simulations. Five simulation results were included in different shades of color.

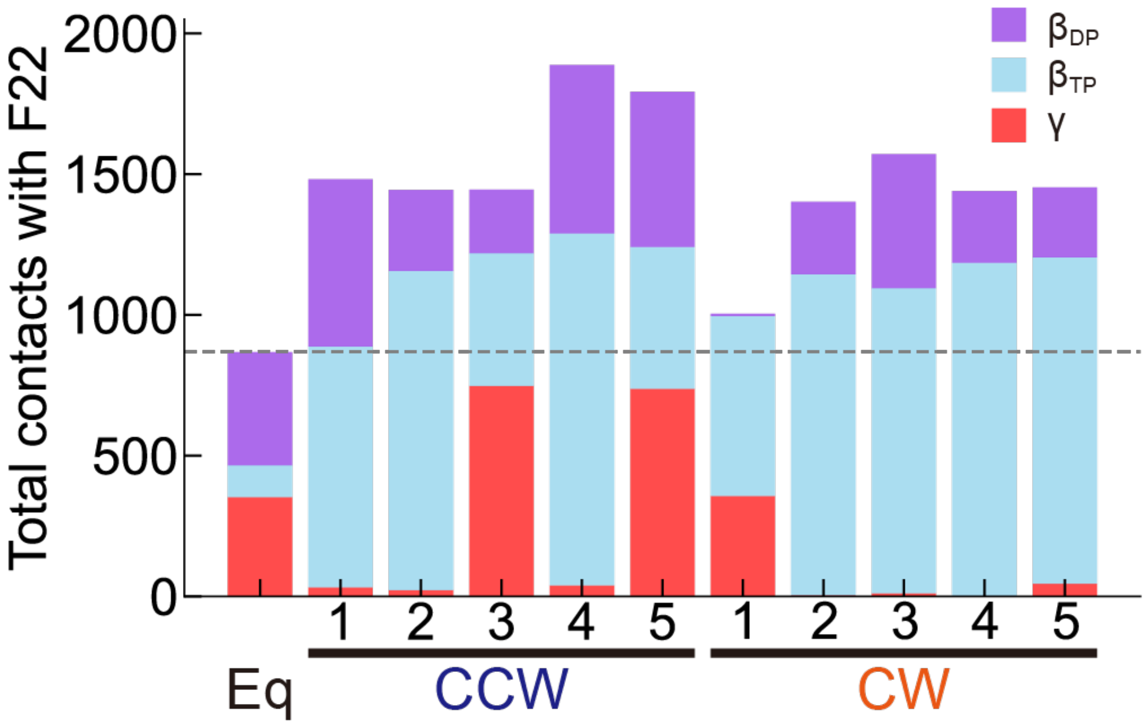

Fig. S9. Contact analysis of F22 from IF$_1$ during the last 30 ns (300 frames) of each simulation. The result of ‘Eq’ represents the simulation result before the torque-applying run (= torque-free run). The dotted line represents the total number of contacts in the ‘Eq’ run as a reference value.

Table. S1. The conformational states and the bound nucleotides in the initial structure, the 1st and 2nd targeted MD.

The $\alpha\beta_E$, $\alpha\beta_{TP}$, and $\alpha\beta_{DP}$ represent the typical conformation of the αβ pair: $\alpha\beta_E$ adopts an open conformation of the β subunit, and the $\alpha\beta_{TP}$ and $\alpha\beta_{DP}$ adopt a closed conformation with its interface loose or tight, respectively. The bound nucleotides are described in the parentheses.

	Initial state	1st targeted MD	2nd targeted MD
$\alpha\beta_1$	$\alpha\beta_{DP}$ (ADP + P_i)	$\alpha\beta_{TP}$ (ATP)	$\alpha\beta_E$ (Empty)
$\alpha\beta_2$	$\alpha\beta_{TP}$ (ATP)	$\alpha\beta_E$ (Empty)	$\alpha\beta_{DP}$ (ADP + P_i)
$\alpha\beta_3$	$\alpha\beta_E$ (Empty)	$\alpha\beta_{DP}$ (ADP + P_i)	$\alpha\beta_{TP}$ (ATP)

References

(1) Okazaki, K.; Hummer, G. Phosphate Release Coupled to Rotary Motion of F_1-ATPase. *Proceedings of the National Academy of Sciences* **2013**, *110* (41), 16468–16473. https://doi.org/10.1073/pnas.1305497110.

(2) Hummer, G.; Szabo, A. Free Energy Reconstruction from Nonequilibrium Single-Molecule Pulling Experiments. *Proceedings of the National Academy of Sciences* **2001**, *98* (7), 3658–3661. https://doi.org/10.1073/pnas.071034098.
